# A novel deep learning-assisted hybrid network for plasmodium falciparum parasite mitochondrial proteins classification

**DOI:** 10.1371/journal.pone.0275195

**Published:** 2022-10-06

**Authors:** Wafa Alameen Alsanousi, Nosiba Yousif Ahmed, Eman Mohammed Hamid, Murtada K. Elbashir, Mohamed Elhafiz M. Musa, Jianxin Wang, Noman Khan, Afnan

**Affiliations:** 1 Department of Computer Science, Faculty of Mathematical and Computer Science, University of Gezira, Wad Madani, Sudan; 2 Preparatory Year Department, Al-Ghad International Colleges for Applied Medical Sciences, Riyadh, Saudi Arabia; 3 Department of Computer Science, College of Science and Arts, Najran University, Sharoura, Saudi Arabia; 4 Department of Information Systems, College of Computer and Information Sciences, Jouf University, Sakaka, Saudi Arabia; 5 Department of Computer Science, College of Computer and Information Sciences, Jouf University, Sakaka, Saudi Arabia; 6 School of Information Science and Engineering, Central South University, Changsha, P.R. China; 7 Sejong University, Seoul, Republic of Korea; Hanyang University, REPUBLIC OF KOREA

## Abstract

Plasmodium falciparum is a parasitic protozoan that can cause malaria, which is a deadly disease. Therefore, the accurate identification of malaria parasite mitochondrial proteins is essential for understanding their functions and identifying novel drug targets. For classifying protein sequences, several adaptive statistical techniques have been devised. Despite significant gains, prediction performance is still constrained by the lack of appropriate feature descriptors and learning strategies in current systems. Moreover, good ground truth data is important for Artificial Intelligence (AI)-based models but there is a lack of that data in the literature. Therefore, in this work, we propose a novel hybrid network that combines 1D Convolutional Neural Network (CNN) and Bidirectional Gated Recurrent Unit (BGRU) to classify the malaria parasite mitochondrial proteins. Furthermore, we curate a sequential data that are collected from National Center for Biotechnology Information (NCBI) and UniProtKB/Swiss-Prot proteins databanks to prepare a dataset that can be used by the research community for AI-based algorithms evaluation. We obtain 4204 cases after preprocessing of the collected data and denote this set of proteins as PF4204. Finally, we conduct an ablation study on several conventional and deep models using PF4204 and the benchmark PF2095 datasets. The proposed model ‘CNN-BGRU’ obtains the accuracy values of 0.9096 and 0.9857 on PF4204 and PF2095 datasets, respectively. In addition, the CNN-BGRU is compared with state-of-the-arts, where the results illustrate that it can extract robust features and identify proteins accurately.

## Introduction

Unicellular eukaryotes or protozoan parasites cause many diseases that impact human health [1]. Malaria is caused by the parasite plasmodium, which kills over one million African children each year. 40% of the worldwide people had been at risk of infection in 2016, as per a World Health Organization (WHO) assessment [2]. The four plasmodium types, which may induce malaria in peoples are Parasite falciparum, Plasmodium vive, Plasmodium malaria, and Plasmodium ovale, with Parasite falciparum being the most hazardous [3]. Plasmodium malaria is found in the salivary cells of female anopheles, and it penetrates the human body to become sporozoites (N). Infection with the malaria parasite occurs when a female anopheles mosquito bites an uninfected person for sporozoites to be injected into the human body [4]. The life cycle of Plasmodium malaria has complicated, comprising two significant cycles: (asexual reproduction) in the human body and (sexual reproduction) in the anopheles mosquito [5]. A prokaryotic cell consists of cytoplasm and a membrane, the mitochondria are membrane-bound organelle within the cytoplasm, moreover the core role of mitochondria is to provide the cell energy needed to control the metabolism and produce Adenosine Triphosphate (ATP) [6] (Table 1 for the list of abbreviations). The mitochondrial proteins of plasmodium falciparum are an important target for anti-malarial medications, and their identification by manual tests is challenging and time-consuming. Mitochondria have their own Deoxyribonucleic Acid (DNA) and ribosomes [7, 8]. However, malaria remains a severe public health hazard despite global attempts to control it, and there is currently no malaria vaccine that any organization has approved [9]. As a result, pharmaceutical institutions must wait for long time to produce their related drugs.

**Table 1:** Abbreviations used in the paper.

DL	Deep Learning	NCBI	National Center for Biotechnology Information
RNN	Recurrent Neural Network	ML	Machine Learning
DL	Deep Learning	MCC	Matthews Correlation Coefficient
NPV	Negative Predictive Value	WHO	World Health Organization
ATP	Adenosine Triphosphate	DNA	Deoxyribonucleic Acid
AI	Artificial Intelligence	PDB	Protein Data Bank
NB	Naive Bayes	FDR	False Discovery Rate
SVM	Support Vector Machine	PCA	Principal Component Analysis
FPR	False Positive Rate	BGRU	Bidirectional Gated Recurrent Unit
NLP	Natural Language Processing	CNN	Convolutional Neural Network
RNN	Recurrent Neural Network	PSSM	Position Specific Scoring Matrix
LR	Logistic Regression	SAAC	Split Amino Acid Composition
FNR	False Negative Rate	GRU	Gated Recurrent Unit
KNN	K-Nearest Neighbors	PAAC	Pseudo Amino Acid Composition
IoT	Internet of Things	DT	Decision Tree

A computationally automated and trustworthy method must be developed to selectively identify proteins, enabling on-time and suitable medication manufacture [10]. By sifting through the vast volumes of complicated data generated throughout the drug discovery process and extracting new and crucial information, AI technologies have the potential to accelerate pharmaceutical research [11]. Other domains, including the diagnosis of malaria based on visual cues, have demonstrated the efficacy of AI approaches. For instance, Loddo et al. [12] compared eleven CNN-based state-of-the-art architectures for malaria analysis using two datasets. Their results illustrated that DenseNet-201 performance was established and robust compared to the other models. Similarly, Abdurrahman et al. [13] fine-tuned object detection models such as YOLOV4 and YOLOV3 for plasmodium detection in profuse blood smear microscopic images. Among all the models, YOLOV4 had attained the highest detection accuracy. In another study, Oyewola et al. [14] introduced a CNN-based model for malaria parasite classification. The model was trained using reinforcement learning strategy and the obtained accuracy was 94.79%.

In this area, a number of computationally sophisticated techniques are being developed, such as Machine Learning (ML) methods for extracting local features from biological sequences and a variety of classifiers to distinguish the different types of proteins. Unfortunately, these methods exhibit mediocre performance and provide non-representative classifiers when attempting to extract contextual features from sequence patterns. Any model must have strong feature engineering in order to deliver accurate results. Feature extraction is carried out manually in ML models. However, when data complexity rises, manual feature selection may result in a number of issues, such as choosing characteristics that don’t provide the optimal answer or omitting crucial features. To solve this problem, automatic feature selection might be employed. According to the literature evaluation, there is a research gap since the generalized model is not used to classify other protein sequences for other disorders. Additionally, reliable ground truth data are crucial for ML and DL models, yet the literature lacks this information. Besides, we notice acceleration in discovering a new number of these proteins. In the year 1995, approximately 50000 (UniProt) and in the year 2021, according to the statistics report of the Protein Data Bank (PDB), it reached 560000 proteins [15]. Finding mitochondrial protein sequences continues to be a challenge. The scholar’s attention is directed to identifying it because the protein sequences represent an essential role in the science of proteins and bioinformatics. The primary contribution of this work may be summed up as follows:

- The most difficult part of sequence classification is feature selection. The situation of high dimensionality is made worse by the most popular representations, because sequences lack specific characteristics. The characteristics may be automatically extracted from the input data using Deep Learning (DL) models, though. As a result, a novel hybrid architecture based on CNN and BGRU is suggested in this research, whereby CNN layers are utilized to extract spatial features and BGRU layers to extract sequential information from the protein sequence while the dense layer serving as a classifier.
- For ML and DL models, reliable ground truth data is crucial, yet the literature lacks this information. In order to classify protein sequences, we thus create a new dataset (PF4204) from multiple data banks, including UniProtKB/Swiss-Prot and NCBI. This information may be used by researchers to ask challenging biological questions and gain additional knowledge and understanding.
- Using the benchmark PF2095 dataset and the PF4204 dataset, we perform an ablation experiment on several ML and DL models. On the PF4204 and PF2095 datasets, the proposed model ‘CNN-BGRU’ yielded accuracy scores of 0.9096 and 0.9857, respectively. The findings demonstrate that our model can reliably identify proteins and extract robust features when it is compared to state-of-the-art approaches.

The remaining sections of the study are structured as follows: Section 2 is devoted to related research, while Section 3 examines the CNN-BGRU architecture that is proposed. Section 4 discusses the significant experimentation, and Section 5 wraps up with the suggested technique.

## Related work

The methods that are used in proteins sequence prediction and categorization of the mitochondrion and non-mitochondrion proteins include ML and DL approaches. The ML-based methods consist of two basic steps: classifier and features. For instance, Wan et al. [16] used a Logistic Regression (LR) classifier to determine protein subcellular localization. Next, to predict protein-protein interaction locations, the Naive Bayes (NB) method was used [17]. Similar to this, Support Vector Machine (SVM), a reliable and effective ML approach, has been extensively utilized to categorize mitochondrial proteins. The basic sequences of proteins may be used to extract characteristics in a variety of ways. For protein sequences of different lengths, techniques for extracting amino acid, dipeptide, and tripeptide characteristics can result in fixed-length data. The method for extracting features that is most frequently employed is called Split Amino Acid Composition (SAAC). For the prediction of mitochondrial transit peptides in Plasmodium falciparum sequences utilizing statistical techniques, Principal Component Analysis (PCA), and supervised neural networks, Bender et al. [18] established an efficient approach named ‘PlasMit’. A novel method dubbed PM-OTC developed by Bian et al. [19] predicts mitochondrial proteins in plasmodium utilizing the chosen tripeptide composition as inputs and SVM as a predictor. In another work, Cai et al. [20] used an SVM-based method to group proteins into functional families in order to learn more about the physicochemical characteristics of various amino acids. In order to predict the mitochondrial proteins of malaria parasites, Verma et al. [21] made a hybrid SAAC, and Position Specific Scoring Matrix (PSSM)-based SVM model. By combining a SAAC and PSSM, Hayat et al. [22] introduced memory-SVM to forecast the kinds of membrane protein. Pseudo Amino Acid Composition (PAAC) and the structural alphabet are used in a study by Zhang et al. [23] to predict the mitochondrial proteins of malaria parasites. To predict the protein’s sub-mitochondrial sites, Zeng et al. [24] used hybrid feature descriptors, PAAC, and pseudo PSSM with SVM classifier. Xiong et al. [25] proposed an ensemble approach for the prediction of type IV secreted effectors of bacteria from the sequence of the proteins. For the classification of mitochondrial proteins, Afridi et al. [26] demonstrated genetic programming and an ensemble strategy based on the feature extraction approach. An ensemble learning-based method for classifying cancer-fighting and non-cancerous peptides was demonstrated by Alsanea et al. [27] in their study.

DL-based methods extract features that help in accessing several biological information, improving prediction accuracy. Some researchers fully harnessed the power of the CNN architecture for applications in computational biology and in modelling the specificity of protein-DNA binding sequences [28]. For instance, Savojardo et al. [29] proposed an effective method called ‘DeepMito’ for protein sub-mitochondrial locations using a CNN. Alipanahi et al. [30] attempt to develop a unique concept in DL to distinguish between DNA-binding and non-DNA-binding proteins is another noteworthy achievement. In addition, Qu et al. [31] used main sequences and two layers CNN, long short-term memory (LSTM) networks to boost learning capacity in their DL-based technique to predicting proteins-DNA binding. To classify the DNA binding proteins, Qiu et al. [28] suggested a novel method based on CNN structures with sequence-based learning. In order to construct malaria parasite vaccines, Su et al. [32] established a very successful model employing DL approaches to categorize mitochondrial and non-mitochondrial proteins. This model provides crucial information for building secure and efficient vaccinations as well as preventing drug resistance. Similarly, Zhang et al. [23] proposed a weakly-supervised CNN-based architecture to predict DNA-binding proteins further their method gave good results compared to other traditional methods.

## The proposed approach

**Fig 1:**
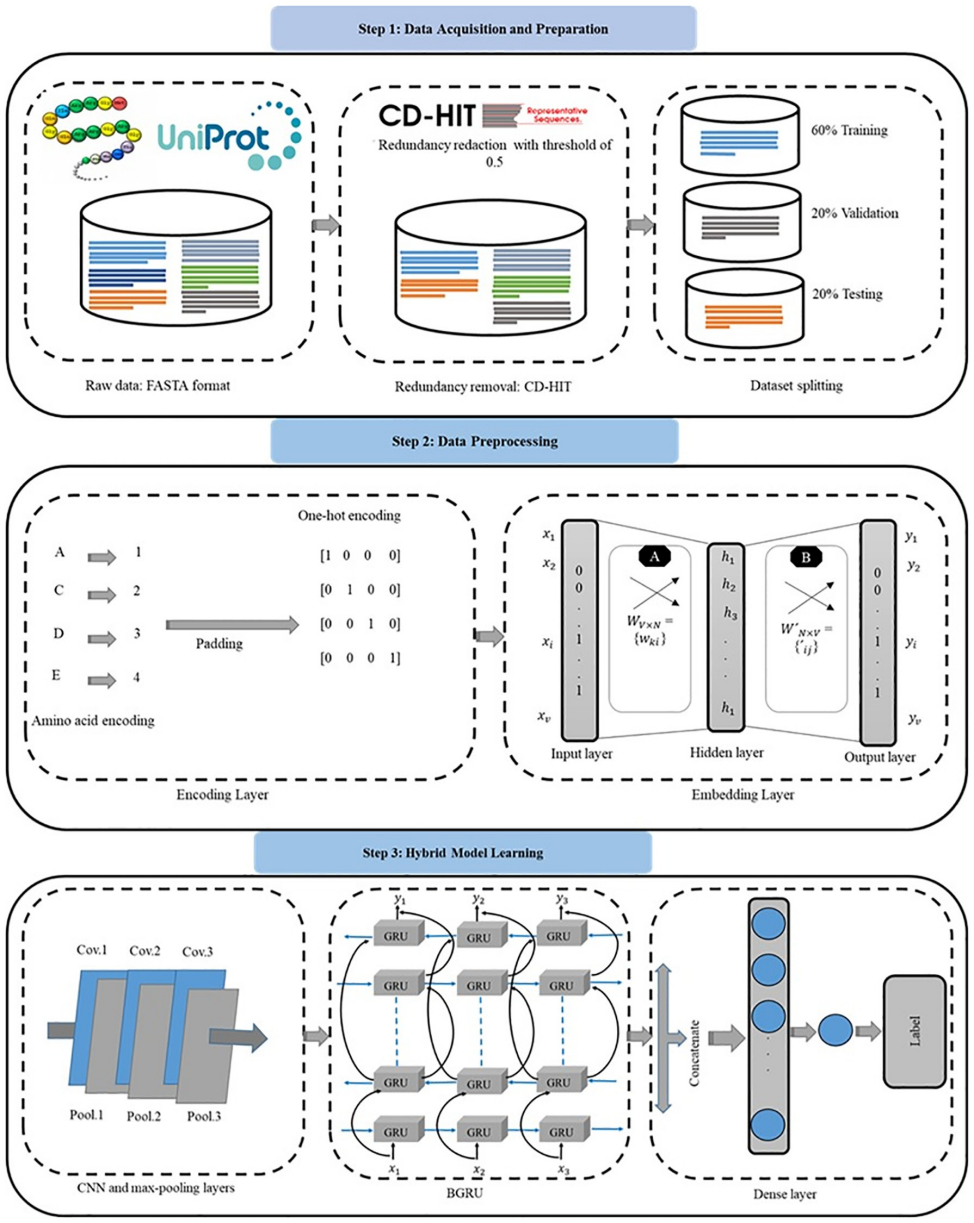
Overview of the proposed hybrid architecture for plasmodium mitochondrial proteins classification.

The entire approach that we used to classify the malaria parasite mitochondrial proteins is depicted in Fig 1 The methodology involves three stages comprising six sub-processes: benchmark dataset collection, data acquisition and preprocessing, protein sequence encoding, embedding layer, CNN, and the sequential model ‘BGRU’. The detailed description of the proposed approach is discussed in the following sub-sections.

### Data acquisition and preparation

The critical factor in constructing mitochondrial protein prediction method of the malaria parasite is to identify suitable datasets. In this study, we utilized a dataset denoted as PF4204 and one existing benchmark dataset PF2095 [10]. The standard dataset PF4204 for this work is obtained from UniProtKB/Swiss-Prot protein databank, which are considered positive samples. The dataset contains 1202 raw sequences in FASTA format. We collected the positive data by searching the keywords: ‘Plasmodium falciparum mitochondrion’ available online [33]. Furthermore, from the NCBI database as positive samples in FASTA format, we collected 1335 raw sequences by searching the keywords ‘mitochondrion Plasmodium falciparum’ and ‘Plasmodium falciparum’ [porgn:__txid5833]) available online [34]. The sources of protein sequences are [RefSeq (3), GenBank (613), DDBJ (690), EMBL (29)]. Moreover, we collected 2445 negative samples by searching the ‘non-mitochondrion proteins’ from UniProtKB/Swiss-Prot protein databank in FASTA format. The PF4204 is a set of protein biological sequences used in this study as a dataset collected from various data banks. The PF4204 contains 4204 samples, 2102 positive (mitochondrion proteins) and 2102 negative (non-mitochondrion proteins) samples. After that, we processed positive and negative sequences using the CD-HIT software [35] with global alignment and a sequence identity threshold of 0.5 to eliminate identical sequences. Then, remove protein sequences with a similarity equal to 80%, remove shorter length (less than 40) of amino acids, and the most extended sequence from each cluster is picked as the final sequence. After the refining process, we collected 2102 and 2102 as the mitochondrion and non-mitochondrion samples, respectively, for classification purposes. There is no need to feed the PF2095 benchmark dataset through the pre-processing phase because it is publicly available, which is downloaded from [10]. The total number of proteins in the dataset is 2095, containing 890 positive (mitochondrion proteins) and 1205 negative (non-mitochondrion proteins) samples. The samples of both datasets are shown in Fig 5(a).

### Data preprocessing

In most protein sequence classification applications, feature encoding is a time-consuming but necessary step in developing a statistical ML model. Various methodologies have presented protein sequence encoding, including homology-based, n-gram, and extraction methods based on physicochemical attributes. Although those methods perform effectively in most situations, their implementation in practice is limited due to the high level of human participation. DL technology’s ability to automatically learn characteristics is one of the most successful parts of its development. In DL models, all input and output variables must be numbers. This implies that before fitting and assessing a model, data must have transformed from categorical data to numerical data. The two most popular methods are one-hot encoding and ordinal encoding. Categorical variables are represented as binary vectors in one-hot encoding. In order to do this, the categorical values must first be converted to integer numbers. The index of the integer, which is denoted with a 1, is then used to represent each integer value as a binary vector with all other values being zero. While it is important to note that the protein sequence encoding has no influence on the outcome, trying to give a regular number to each amino acid results in the encoding technique essentially creating a digital vector of a protein sequence with a predetermined length. In Natural Language Processing (NLP), words are represented using the vector space model. Each discrete vocabulary word is embedded into a continuous vector space using the mapping technique known as embedding. In this method, semantically related phrases are mapped to semantically related places. This is done by adding a weight matrix *W𝜖 R*^*d X|V|*^ to the one-hot vector from the left, where *|V|* is the number of distinct symbols in the lexicon as stated in Eq 1. After the embedding layer, the input amino acid sequence is transformed into a series of dense real-valued vectors, like as (*V1*, *V2*…*Vt*). Consider that each number mapping has a fixed vector length, and that the dense output vector length is now *8x1* in the embedding layer. Layer proteins will cause the sequence to transform into an *8x8* matrix.


$$V_{t}=W_{X_{t}}$$ (1)

### Hybrid model learning

This section discusses the proposed hybrid network for plasmodium falciparum parasite mitochondrial proteins classification.

#### Convolutional neural network

The DL algorithms operate very effectively in classification by extracting feature representations dependent on convolution and the max-pooling layer. The CNN is a powerful DL approach. Besides, a CNN is a deep feed-forward network that extends a traditional artificial neural network by adding extra layers and convolutional blocks. Implementing convolution blocks in the network gave rise to ‘convolutional’. A CNN structure comprises three layers: convolutional, pooling, and fully connected [36]. Convolutions, activation functions, pooling, dropout, batch normalization, fully linked blocks, and other techniques are used in these layers, coupled in various ways. The following aspects can be used to construct a CNN structure: The input data can be in one of three formats: one-dimensional (1D), two-dimensional (2D), or three-dimensional (3D). This data can come from numerous sources such as sensors, audio, video, and 3D images. Next the convolutional layers can accomplish the feature extraction tasks. The extraction is processed by using convolution operations to the input, and the outcome is sent to the following layer’s input [37]. Several filters, kernels, padding, and stride are used to design the convolutions operations, which result in a feature map after implementing an activation function like *ReLU* or *tanh*. Furthermore, the pooling layers, which are generally practiced after a convolutional layer, are in charge of storing the information created by the feature maps. For example, when processing an image, these layers substantially decrease the input amount, reduce calculation times, speed up the training process, and result in more accurate features extraction. Max pooling is the most popular strategy used on these layers. Therefore, the fully connected layer is a traditional back-propagation neural network that handles the features produced by the previous layers. It generates a prediction for the network’s final output, a regressing task, such as a metric forecast, or a classification task, such as classifying an image.

This research utilized a 1D CNN because there are many benefits to using 1D CNNs, including great results on smaller sample sizes, easy implementation compared to 2D CNNs and other DL structures, a faster training process and excessive extracting important features from sequence data and time-series [38]. Since the data displays a matrix structure, we start giving input data to the model. First, compose the data in a fixed-size matrix and send it to the convolution layer for image-like processing when dealing with gene encoding. The framework in Fig 1 shows that the convolution layers are used to extract the features and pooling layers accompany each one to extract deep and important features. For the convolution layer to process the data, mitochondrial protein sequence is generated in a matrix of constant size. Convolution layers make up the model in this study, and each is followed by a max-pooling layer for the extraction of important patterns. A filter is needed to check the amino-acid sequences throughout this layer to receive a new function map.

#### Gated recurrent units network

**Fig 2:**
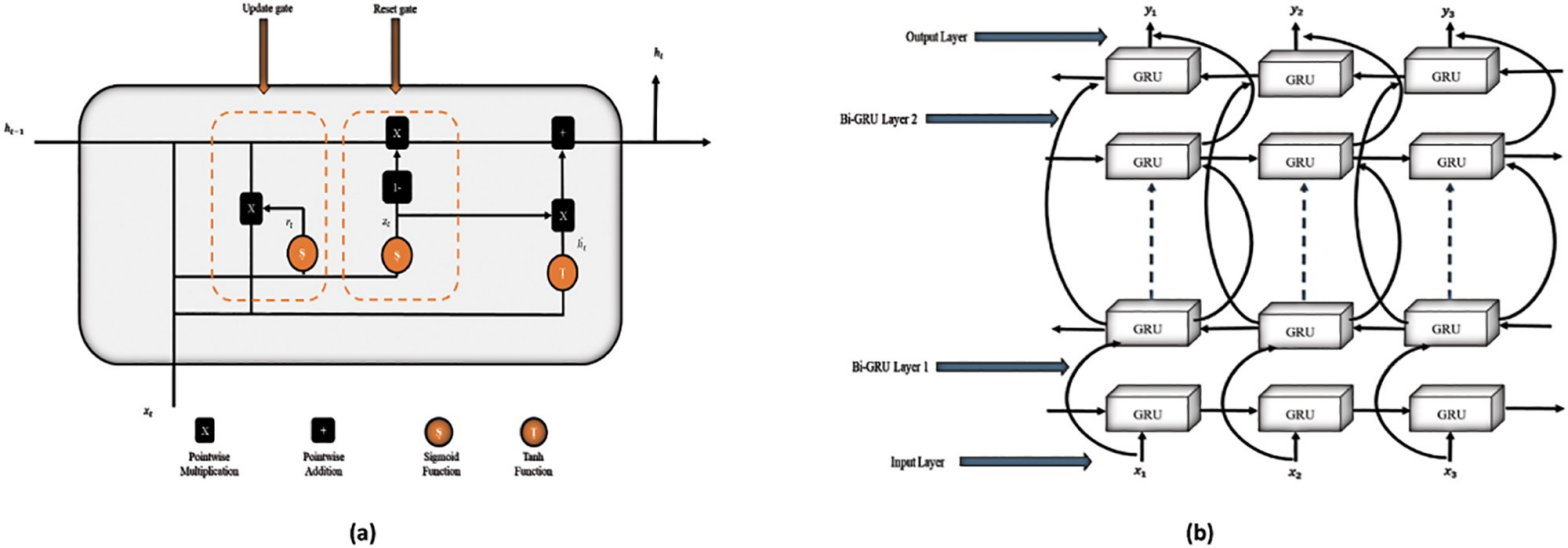
(a) represents unit structure of GRU while (b) shows the working flow of BGRU.

GRU is a form of sequential models that addresses the problem of long-term dependencies, which can cause vanishing gradients in more extensive vanilla neural networks. GRU solves this problem by storing memory from prior time points to assist the network make better predictions in the future. GRU emphasizes the construction of gates, which control information processing and storage and consent the network’s hidden states to be updated and forgotten. The internal structure review of GRU contains the update gate, which determines what data to remove and what different material to include, while the reset gate determines how much prior knowledge to forget [39]. In Fig 2(a), *z*_*t*_ presents the update gate, *r*_*t*_ denotes the reset gate, $h_{t}^{\sim }$ represents the applicant hidden state of the currently hidden node, *h*_*t*_ represents the current hidden state, *x*_*t*_ represents the current neural network’s input, and *h*_*t*−1_ represents the previously hidden state. The following is the whole calculating Eqs (2–5). Where *σ* is the sigmoid activation function, which can be range between 0 and 1. Which determines the relevance of previous information and then applies it to the contender for the updated value. Where ⊙ the matrix’s Hadamard product, matrices of weights are *u* and *w*, *z*_*t*_ and *r*_*t*_ are ranging between 0 to 1. The current cell state *h*_*t*_ is the result of filtering the previous cell state *h*_*t*−1_ and the updated candidate $h_{t}^{\sim }$. The update gate *z*_*t*_ determines the amount of updated candidates required to calculate the current cell state and the amount of previous cell state that is maintained [40].


$$z_{t=\sigma \;(w_{zx}\;x_{t}\;+\;u_{zh\;}h_{t-1})}$$ (2)

$$r_{t=\sigma \;\left(w_{rx}\;x_{t}\;+\;u_{rh\;}h_{t-1}\right)}$$ (3)

$$h_{t}^{\sim }=tan\;\left(w_{hx}x_{t}+r_{t}\;⊙\;u_{hh}h_{t-1}\right)$$ (4)

$$h_{t}=\left(1-z_{t}\right)⊙\;h_{t}^{\sim }+\;z_{t}\;⊙\;h_{t-1}$$ (5)

#### Multi-layer bidirectional gated recurrent units network

A BGRU is an updated form of GRU, which follows a two-layer topology. Therefore, this layout provides the output layer with all contextual information from the input layer at any given moment [41]. The basic idea behind the BGRU is that the input sequence is processed through a forward and backward direction, with both outputs connected in the same output layer. The basis working flow of BGRU is displayed in Fig 2(b). In the BGRU neural network, the forward layer computes the output of the hidden layer at each step from forward to backward, while the backward layer does the opposite. The output layer superimposes and normalizes the output results of the forward and backward layers at any given time, where *h*_*t*_ is the output vector of the hidden layer of the forward layer in the first and second layers of the BGRU neural network at time *t*, and *h*_*t*_ is the output vector of the hidden layer of the backward layer in the first and second layers of the BGRU neural network at time *t*, and *x*_*t*_ is the neural network input at time *t*. The concatenation of the forward and backward outputs is indicated by the Eq (8), in this case.


$$h_{tf}=f\left(w_{1}\;x_{t}+\;w_{2}\;h_{t-1}+\;b\right)$$ (6)

$$h_{tb}=f\left(w_{3}\;x_{t}+\;w_{4}\;h_{t-1}+\;b\right)$$ (7)

$${\;o}_{t}=\left(h_{tf},\;h_{tb}\right)$$ (8)

## Experimental results

The specifics of the system configuration and implementation, evaluation metrics, and result comparison of several models are covered in this section, which is about the experimental outcomes.

### System configuration and implementation details

The sequential models for classifying Plasmodium mitochondrial proteins are implemented in Python (3.8.5, using Keras (2.5.0) with Tensorflow (2.5.0) as the backend. The hardware setup consists of a Windows 10 operating system with a Processor Intel^®^ Core^™^ i7-9750H CPU @ 2.60GHz, NVIDIA graphics processing unit (GeForce GTX 950), and 16.0 GB installed RAM. Two separate proteins datasets, PF4204 and PF2095 [10], are utilized to confirm and validate the efficiency of the different sequential models. The models are then validated using the hold-out and k-fold cross-validation approaches. The hold-out approach is used when the data is fragmented into training, validation, and testing sets. While the training set and validation set are used to train the model and validate it during training, the test set is used to assess how well the model performs on data that has not yet been seen. In this study, a typical split of data is considered using the hold-out method, which is to use 60% of the data for training, 20% for validation, and the final 20% for testing. Similarly, the dataset is randomly partitioned into ’*k*’ groups for k-fold cross-validation. One of the groups is used for the test set, while the other groups are used for the training set. After being trained on the training set, the model is put to the test. The method is then carried out again after using each different group as the test set. In this study 10-fold cross-validation is used where the dataset is split into 10 groups, the model is trained and tested 10 times in total, and each group has the chance to act as the test set.

It is difficult to determine a hyperparameter and various interactions between hyperparameters for a certain dataset. A better strategy is to systematically compare various choices for model hyperparameters and select the subset that produces a model that performs the best on a particular dataset. Hyperparameter optimization is the term used for this. One collection of effective hyperparameters that may be used to design a model is the outcome of a hyperparameter optimization. Hyperparameters are configurable settings that let a ML or DL model be tuned for a particular task. In this study we used grid search optimization method for the proposed model hyperparamets values setting. Grid search analyzes each place in the grid and defines a search space as a grid of hyperparameter values. Grid search is excellent for spotting combinations that consistently produce good results. Adam optimizer is used to train each model for up to 50 epochs on the dataset, using a learning rate of 0.001 and a batch size of 16. Furthermore, two 1D CNN layers with 64 and 32 filters, 6 and 3 kernel size are used where each layer is followed by pooling layer. Next two BGRU layers are used with 30 and 20 units followed by a dense layer with 20 neurons. For the proposed model, overfitting is controlled using k-fold cross-validation, regularization, dropout, and early stopping mechanism.

### Evaluation metrics

The proposed network is evaluated using a variety of assessment metrics. These include accuracy, False Discovery Rate (FDR), sensitivity, False Negative Rate (FNR), precision, False Positive Rate (FPR), specificity, f1 score, Negative Predictive Value (NPV), and Matthews Correlation Coefficient (MCC). The mathematical formulas are defined in Eqs (9)–(18).


$$Accuracy=\frac{\left(TP\;+\;TN\right)}{\left(P\;+\;N\right)}$$ (9)

$$Sensitivity=\frac{TP}{TP+FN}$$ (10)

$$Specifictiy=\frac{TN}{TN+FP}$$ (11)

$$Precision=\frac{TP}{TP+FP}$$ (12)

$$F1\;Score=\frac{2TP}{2TP+FP+\;FN}$$ (13)

$$MCC=\frac{TP*TN-FP*FN}{\sqrt{\left(TP+FP)(TP+FN)(TN+FP)(TN+FN\right)}}$$ (14)

$$NPV=\frac{TN}{\left(TN+FN\right)}$$ (15)

$$FPR=\frac{FP\;}{(TN\;+\;FP)\;}$$ (16)

$$FDR=\frac{FP}{\left(FP\;+\;TP\right)}$$ (17)

$$FNR=\frac{FN}{\left(FN\;+\;TP\right)}$$ (18)

Where TP represents the proportion of correctly predicted mitochondrial proteins, FP represents the proportion of incorrectly predicted non-mitochondrial proteins, TN represents the proportion of correctly predicted non-mitochondrial proteins, and FN represents the proportion of incorrectly predicted mitochondrial proteins.

### Ablation study on the PF4204 dataset

The overall results obtained on the PF4204 dataset are discussed in this section. Two methods of evaluation are performed on the PF4204 dataset hold-out and 10-fold cross-validation methods, which use both ML and DL algorithms. In hold-out method, 60% of the total samples in this dataset are utilized for training, 20% for validation, while the remaining 20% are used for model testing. We perform ablation study to assess the performance of different ML and DL models. In order to evaluate the prediction performance of the ML models using the hold-out evaluation method. We compare five commonly used ML algorithms including LR, NB, K-Nearest Neighbor (KNN), Decision Tree (DT), and SVM. First, the LR algorithm achieved an accuracy of 0.7848, while NB achieved 0.7979, also checked the KNN, DT, and SVM algorithms, where the SVM outperformed the rest of the algorithms with an accuracy of 0.8216. Employing the 10-fold cross-validation assessment approach. The LR algorithm’s accuracy was 0.7363, while the NB algorithm’s accuracy was 0.7530. We also looked at the KNN, DT, and SVM algorithms, with the SVM outperforming the others with the accuracy of 0.8005. Different evaluation metrics values of each ML algorithm using hold-out and 10-fold cross validation are presents in the Tables 2 and 3, respectively.

**Table 2:** Performance of different models on PF4204 dataset using hold-out validation method.

Metrics/Models	LR	NB	KNN	DT	SVM	GRU	BGRU	CNN-GRU	Proposed
Sensitivity	0.7816	0.7936	0.7986	0.8050	0.8165	0.8506	0.8736	0.8956	0.9070
Specificity	0.7882	0.8025	0.8094	0.8148	0.8272	0.8621	0.8867	0.9024	0.9124
Precision	0.7981	0.8122	0.8192	0.8239	0.8357	0.8685	0.8920	0.9061	0.9155
NPV	0.7711	0.7831	0.7880	0.7952	0.8072	0.8434	0.8675	0.8916	0.9036
FPR	0.2118	0.1975	0.1906	0.1852	0.1728	0.1379	0.1133	0.0976	0.0876
FDR	0.2019	0.1878	0.1808	0.1761	0.1643	0.1315	0.1080	0.0939	0.0845
FNR	0.2184	0.2064	0.2014	0.1950	0.1835	0.1494	0.1264	0.1044	0.0930
F1 Score	0.7898	0.8028	0.8088	0.8144	0.8260	0.8595	0.8827	0.9008	0.9112
MCC	0.5695	0.5957	0.6076	0.6195	0.6433	0.7123	0.7599	0.7979	0.8192
Accuracy	0.7848	0.7979	0.8038	0.8098	0.8216	0.8561	0.8799	0.8989	0.9096

**Table 3:** Performance of different models on PF4204 dataset using 10-fold cross-validation method.

Metrics/Models	LR	NB	KNN	DT	SVM	GRU	BGRU	CNN-GRU	Proposed
Sensitivity	0.7469	0.7582	0.7642	0.7755	0.7967	0.8347	0.8506	0.8607	0.8921
Specificity	0.7222	0.7458	0.7600	0.7727	0.8057	0.8436	0.8611	0.8870	0.9167
Precision	0.7826	0.8043	0.8174	0.8261	0.8522	0.8783	0.8913	0.9130	0.9348
NPV	0.6806	0.6911	0.6963	0.7120	0.7382	0.7906	0.8115	0.8220	0.8639
FPR	0.2778	0.2542	0.2400	0.2273	0.1943	0.1564	0.1389	0.1130	0.0833
FDR	0.2174	0.1957	0.1826	0.1739	0.1478	0.1217	0.1087	0.0870	0.0652
FNR	0.2531	0.2418	0.2358	0.2245	0.2033	0.1653	0.1494	0.1393	0.1079
F1 Score	0.7643	0.7806	0.7899	0.8000	0.8235	0.8559	0.8705	0.8861	0.9130
MCC	0.4662	0.4997	0.5190	0.5432	0.5964	0.6735	0.7073	0.7413	0.8037
Accuracy	0.7363	0.7530	0.7625	0.7743	0.8005	0.8385	0.8551	0.8717	0.9026

**Fig 3:**
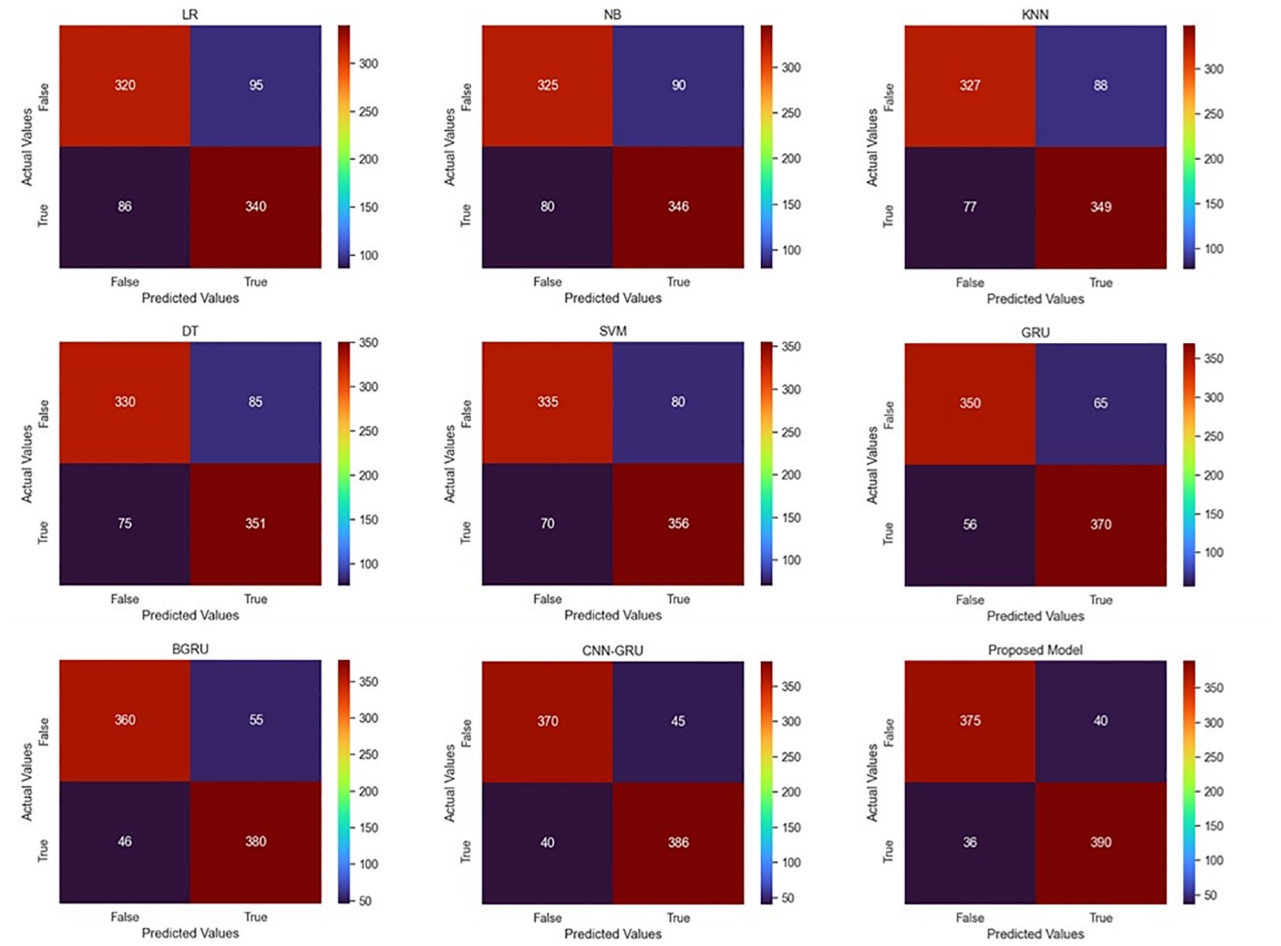
Comparative confusion matrices of different models using PF4204 dataset and hold-out validation method.

Using the hold-out assessment approach, the prediction performance of the DL models is also assessed. The performance of GRU achieved 0.8561 accuracy. Similarly, the BGRU network is also studied to classify mitochondrial proteins for the plasmodium falciparum parasite. The performance obtained by the BGRU is 0.8799 accuracy. The next model is CNN and GRU-based hybrid connection network that shows better performance than previous models, which obtains 0.8989 accuracy. The proposed model used CNN with the integration of BGRU and achieved 0.9096 accuracy. Employing the 10-fold cross-validation assessment approach. The performance of GRU achieved 0.8385 accuracy. Similarly, the BGRU network is also studied to classify mitochondrial proteins for the plasmodium falciparum parasite. The performance obtained by the BGRU is 0.8551 accuracy. The next model CNN and GRU-based hybrid connection network showed better performance than previous models, which obtained 0.8717 accuracy. Finally, the proposed model obtained value of accuracy is 0.9026. The detailed comparative results for ML and DL using both types of evaluation methods are presented in Tables 2 and 3 while the confusion matrices of all the models are shown in Fig 3.

### Ablation study on the PF2095 dataset

The overall results obtained on the PF2095 dataset are discussed in this section. Same strategy is applied to evaluate all the model as for PF4204 dataset is used. To evaluate the prediction performance of the ML models using the hold-out evaluation method, five commonly used ML algorithms are used. First, the LR algorithm achieved an accuracy of 0.7518, while NB achieved 0.7971, also checked the KNN, DT, and SVM algorithms, where the SVM outperformed the rest of the algorithms with the accuracy of 0.8592. Employing the 10-fold cross-validation assessment approach. The LR algorithm’s accuracy was 0.7857, while the NB algorithm’s accuracy was 0.8143. We also looked at the KNN, DT, and SVM algorithms, where the SVM outperforming the others with an accuracy of 0.8476.

**Fig 4:**
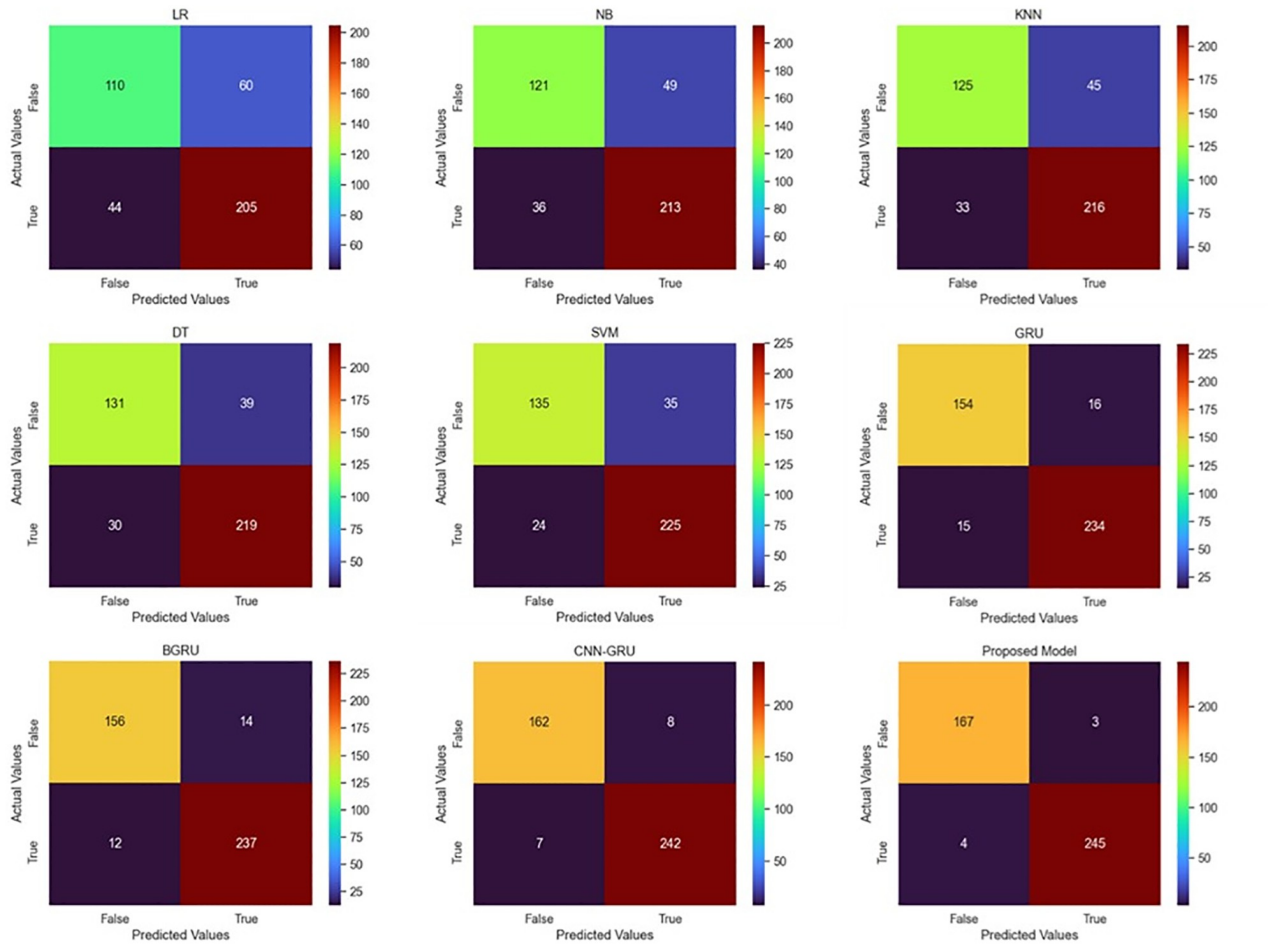
Comparative confusion matrices of different models using PF2095 dataset and hold-out validation method.

Using the hold-out assessment approach, the prediction performance of the DL models is also assessed. The performance of GRU achieved 0.9260 accuracy. Similarly, the BGRU network is also studied to classify mitochondrial proteins for the Plasmodium falciparum parasite. The performance obtained by the BGRU is 0.9379 accuracy. The next model CNN and GRU-based hybrid connection network showed better performance than previous models, which obtained 0.9642 accuracy. Finally, the proposed model achieved 0.9833 accuracy. Employing the 10-fold cross-validation assessment approach. The performance of GRU achieved 0.9143 accuracy. Similarly, the BGRU network is also studied to classify mitochondrial proteins for the Plasmodium falciparum parasite. The performance obtained by the BGRU is 0.9238 accuracy. The next model is a hybrid connection CNN and GRU and showed better performance than previous models, which obtained 0.9476 accuracy while the proposed model obtained value of accuracy is 0. 9857. The detailed comparative results for ML and DL using both types of evaluation methods are presented in Tables 4 and 5 while the confusion matrices are shown in Fig 4.

**Table 4:** Performance of different models on PF2095 dataset using hold-out validation method.

Metrics/Models	LR	NB	KNN	DT	SVM	GRU	BGRU	CNN-GRU	Proposed
Sensitivity	0.7736	0.8130	0.8276	0.8488	0.8654	0.9360	0.9442	0.9680	0.9879
Specificity	0.7143	0.7707	0.7911	0.8137	0.8491	0.9112	0.9286	0.9586	0.9766
Precision	0.8233	0.8554	0.8675	0.8795	0.9036	0.9398	0.9518	0.9719	0.9839
NPV	0.6471	0.7118	0.7353	0.7706	0.7941	0.9059	0.9176	0.9529	0.9824
FPR	0.2857	0.2293	0.2089	0.1863	0.1509	0.0888	0.0714	0.0414	0.0234
FDR	0.1767	0.1446	0.1325	0.1205	0.0964	0.0602	0.0482	0.0281	0.0161
FNR	0.2264	0.1870	0.1724	0.1512	0.1346	0.0640	0.0558	0.0320	0.0121
F1 Score	0.7977	0.8337	0.8471	0.8639	0.8841	0.9379	0.9480	0.9699	0.9859
MCC	0.4790	0.5754	0.6107	0.6563	0.7060	0.8464	0.8711	0.9257	0.9654
Accuracy	0.7518	0.7971	0.8138	0.8353	0.8592	0.9260	0.9379	0.9642	0.9833

**Table 5:** Performance of different models on PF2095 dataset using 10-fold cross-validation method.

Metrics/Models	LR	NB	KNN	DT	SVM	GRU	BGRU	CNN-GRU	Proposed
Sensitivity	0.8818	0.9091	0.9189	0.9279	0.9292	0.9744	0.9829	0.9758	0.9922
Specificity	0.6800	0.7100	0.7273	0.7374	0.7526	0.8387	0.8495	0.9070	0.9756
Precision	0.7519	0.7752	0.7907	0.7984	0.8140	0.8837	0.8915	0.9380	0.9845
NPV	0.8395	0.8765	0.8889	0.9012	0.9012	0.9630	0.9753	0.9630	0.9877
FPR	0.3200	0.2900	0.2727	0.2626	0.2474	0.1613	0.1505	0.0930	0.0244
FDR	0.2481	0.2248	0.2093	0.2016	0.1860	0.1163	0.1085	0.0620	0.0155
FNR	0.1182	0.0909	0.0811	0.0721	0.0708	0.0256	0.0171	0.0242	0.0078
F1 Score	0.8117	0.8368	0.8500	0.8583	0.8678	0.9268	0.9350	0.9565	0.9883
MCC	0.5764	0.6352	0.6627	0.6823	0.6983	0.8297	0.8494	0.8918	0.9700
Accuracy	0.7857	0.8143	0.8286	0.8381	0.8476	0.9143	0.9238	0.9476	0.9857

### Comparative analysis and discussion using PF2095 dataset

**Fig 5:**
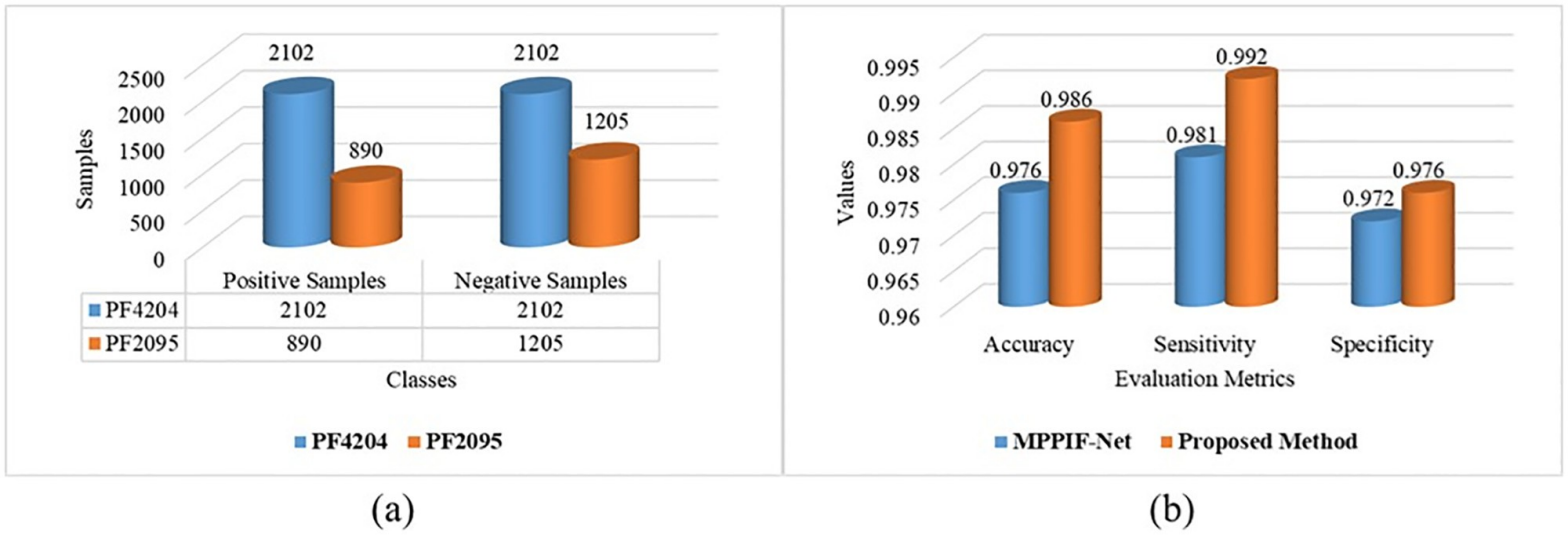
(a) statistics of the proteins datasets, (b) comparative analysis with state-of-the-art model.

This section illustrates the proposed architecture’s comparison to the competitive state-of-the-art method in detail. The comparison is made using the PF2095 dataset and several evaluation metrics. For instance, MPPIF-Net is a hybrid model [10] that used encoder-decoder type architecture for proteins sequence classification. The encoder part of the network is used to extract the feature while decoder part learns these features and classifies the input proteins sequence. Some evaluation metrics are used to predict the plasmodium mitochondrial proteins, with an overall of 0.976 accuracy, 0.981 sensitivity, and 0.972 specificity. Finally, the proposed model achieves 0.9857 accuracy, 0.9922 sensitivity, and 0.9756 specificity, proving that the proposed model outperforms the state-of-the-art model. Fig 5(b) shows the comparative results in more detail. The main drawback of our model is the using of fixed length protein sequence as input. Further, in this study we focus only on binary classification.

## Conclusion and future research direction

Identification of mitochondria proteins of plasmodium plays an important role to discover anti-malaria drug targets, thus reducing mortality. This work designed a novel 1D and BGRU-based hybrid architecture to classify the plasmodium falciparum parasite mitochondrial proteins sequences. The proposed network is capable to swiftly and effectively distinguishing proteins and automatically extracting deep features. It improves the prediction accuracy as well as the fitting of uncharacterized data. In addition, the proteins sequences (PF4204) dataset is collected from UniProt databank in the FASTA format and then preprocessed. Two datasets, the PF4204 and PF2095 datasets, as well as nine different ML and DL models, GRU, BGRU are utilized to validate and assess the proposed model’s efficacy. The comparison analysis reveals that our model is both effective and efficient, with good classification accuracy. On the FP4204, the proposed model achieves 90.96% accuracy. Similarly, the model obtained 98.57% accuracy, on the FP2095 dataset. Finally, the suggested framework improves prediction accuracy as well as the fitting of uncharacterized data. In the future, we will explore the fusion of traditional and deep features using ensemble learning approach to improve the performance. Further, considering the importance of Internet of Things (IoT), light weight will be illustrated that can be run easily on resource-constrained devises.

## Supporting information

S1 Dataset (CSV)
